# Therapeutic Targets in Chlamydial Fatty Acid and Phospholipid Synthesis

**DOI:** 10.3389/fmicb.2018.02291

**Published:** 2018-09-25

**Authors:** Jiangwei Yao, Charles O. Rock

**Affiliations:** Department of Infectious Diseases, St. Jude Children’s Research Hospital, Memphis, TN, United States

**Keywords:** Chlamydia, fatty acid synthesis, phospholipid, enoyl-ACP reductase, antibiotic target

## Abstract

*Chlamydia trachomatis* is an obligate intracellular pathogen with a reduced genome reflecting its host cell dependent life style. However, *C. trachomatis* has retained all of the genes required for fatty acid and phospholipid synthesis that are present in free-living bacteria. *C. trachomatis* assembles its cellular membrane using its own biosynthetic machinery utilizing glucose, isoleucine, and serine. This pathway produces disaturated phospholipid molecular species containing a branched-chain 15-carbon fatty acid in the 2-position, which are distinct from the structures of host phospholipids. The enoyl reductase step (FabI) is a target for antimicrobial drug discovery, and the developmental candidate, AFN-1252, blocks the activity of *Ct*FabI. The x-ray crystal structure of the *Ct*FabI•NADH•AFN-1252 ternary complex reveals the interactions between the drug, protein, and cofactor. AFN-1252 treatment of *C. trachomatis*-infected HeLa cells at any point in the infection cycle reduces infectious titers, and treatment at the time of infection prevents the first cell division. Fatty acid synthesis is essential for *C. trachomatis* proliferation within its eukaryotic host, and *Ct*FabI is a validated therapeutic target against *C. trachomatis*.

## Introduction

*Chlamydia trachomatis* is an obligate intracellular bacterial parasite that causes a range of human diseases including trachoma and sexually transmitted infections (Belland et al., 2004). In the pre-genomic age, *Chlamydia* was thought to be an “energy parasite” because they imported ATP from the host cell (Hatch et al., 1982; Iliffe-Lee and McClarty, 1999), and it was unclear which other major metabolic pathways *C. trachomatis* encoded endogenously and which pathways *C. trachomatis* depended on the host (Omsland et al., 2014). The publication of the first *C. trachomatis* genome was reported in 1998, and showed an organism with a reduced genome (ca. 1 million bp) that encoded the major metabolic pathways (glycolysis, DNA synthesis, RNA synthesis, protein synthesis, and phospholipid synthesis) found in free-living bacteria, but lacked many genes necessary to synthesize the precursors to these pathways (Stephens et al., 1998). Instead, *C. trachomatis* encoded a variety of pumps to acquire these precursors such as amino acids, nucleotides, and cofactors from the host (Braun et al., 2008; Bastidas et al., 2013). The genome sequence of *C. trachomatis* paints the picture of a free-living bacteria that adapted to intracellular growth by stealing metabolic precursors instead of synthesizing them endogenously, but retained the major metabolic pathways.

**FIGURE 1:**
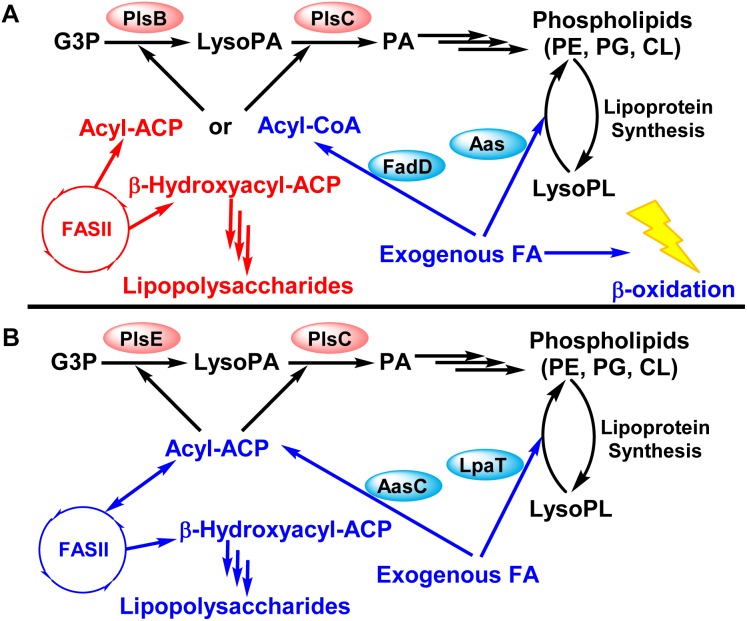
Schematic comparison of phospholipid synthesis in *Escherichia coli* (free living bacteria) and *Chlamydia trachomatis* (obligate intracellular bacteria). Modified from Yao and Rock (2017b). **(A)** Phospholipid synthesis pathway in *E. coli*. **(B)** Phospholipid synthesis in *C. trachomatis*. *C. trachomatis* encodes all the genes necessary for type II fatty acid (FASII) and phospholipid synthesis from acetate, glycerol-3-phosphate, serine, and isoleucine typically found in free-living bacteria. Fatty acids are made in the type II fatty acid synthesis system (FASII). Two acyltransferases (PlsE and PlsC in *C. trachomatis* vs. PlsB and PlsC in *E. coli*) make phosphatidic acid (PA). Phosphatidic acid is the precursor to the various phospholipid species, and *C. trachomatis* encode for the genes to synthesize phosphatidylethanolamine (PE), phosphatidylglycerol (PG), and cardiolipin (CL) like *E. coli*. Exogenous fatty acids are converted into acyl-CoA in *E. coli*. Acyl-CoA cannot be elongated by FASII, and can only be used by the acyltransferases or broken down by β-oxidation. In contrast, exogenous fatty acids are converted into acyl-ACP in *C. trachomatis*, which can be elongated by the FASII or used by the acyltransferases. *C. trachomatis* is not predicted to encode for β-oxidation genes.

**FIGURE 2:**
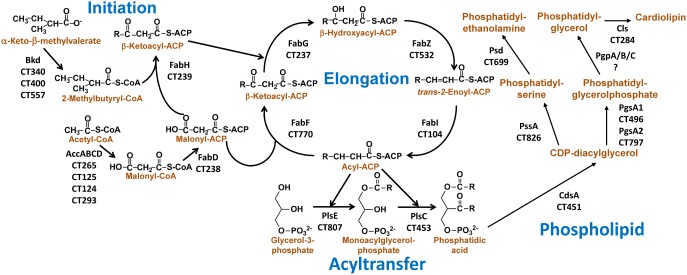
Detailed diagram for fatty acid and phospholipid synthesis in *C. trachomatis*. From Yao et al. (Yao et al., 2015a). This figure shows the protein name designations along with the *C. trachomatis* gene locus number for each reaction. The biosynthetic pathway is divided into four modules. The initiation module consists of the enzymes that supply the precursors to initiate FASII. The elongation module sequentially elongates the acyl chain. The acyltransfer module extracts acyl-ACP from the elongation cycle to acylate glycerol-phosphate. The phospholipid module diversifies the headgroups to produce PE, PG, and CL. Despite a few minor quirks such as the plastidial related enoyl-ACP reductase (FabI) and 1-acyl-*sn*-glycerol-3-phosphate acyltransferase (PlsE), fatty acid and phospholipid synthesis in *C. trachomatis* is the same as other free-living bacteria such as *E. coli* and *Staphylococcus aureus*.

*Chlamydia trachomatis* encodes all the genes necessary to endogenously synthesize phospholipid species typically found in free-living bacteria from acetate, glycerol-3-phosphate, isoleucine, and serine (Yao et al., 2015a) (**Figures 1**, **2**). However, early research into *C. trachomatis* phospholipid synthesis suggested that *C. trachomatis* synthesized phospholipids by modifying host phospholipids (Wylie et al., 1997; Su et al., 2004). Because fatty acid and phospholipid synthesis are targets for antibiotic development, extensive efforts were undertaken to characterize the dependence of *C. trachomatis* on *de novo* phospholipid synthesis and host fatty acid incorporation (Yao et al., 2014, 2015a,b). In this review, we summarize the experimental evidence that *C. trachomatis* produces the bulk of their membrane phospholipid via their encoded fatty acid and phospholipid biosynthetic pathways. Although *C. trachomatis* is capable of activating host fatty acids using an acyl-acyl carrier protein (ACP) synthetase, FASII is essential for *Chlamydia* proliferation, and we evaluate the potential for targeting this pathway with contemporary FASII therapeutics.

## Fatty Acid and Phospholipid Synthesis in *C. trachomatis*

*Chlamydia trachomatis* has a dimorphic life cycle (Abdelrahman and Belland, 2005). The infectious elementary body is responsible for the spread of the infection by attaching and invading susceptible cells. The elementary body is internalized in membrane bound vacuoles, called chlamydial inclusions, and differentiates into the metabolically active reticulate body. The reticulate body undergoes logarithmic growth within the chlamydial inclusions, which expands as the number of reticulate bodies increase. Reticulate bodies directly associate and interact with the chlamydial inclusion using the inclusion membrane proteins (Mirrashidi et al., 2015; Weber et al., 2017). Reticulate bodies differentiate back into elementary bodies, and the lysis of the infected host cell releases the elementary bodies into the environment for further spread and infections.

A major challenge in understanding *C. trachomatis* phospholipid synthesis is deciphering the contribution of bacterial phospholipid synthesis vs. host phospholipid acquisition in the replication and the life cycle of *C. trachomatis*. Research has shown that both host and bacterial lipid synthesis is essential for the life cycle of *C. trachomatis*. *C. trachomatis* encodes for a fully functional fatty acid and phospholipid synthesis system that is analogous to free living bacteria such as *Escherichia coli* (**Figure 1**), and this review will start with the endogenous synthesis of fatty acids and phospholipids.

### Type II Fatty Acid Synthesis in *C. trachomatis*

The genome of *C. trachomatis* is predicted to encode for a complete type II bacterial fatty acid synthesis (FASII) system capable of producing branched-chain fatty acids (**Figure 2**, Initiation and Elongation) (Bidawid et al., 1989; Stephens et al., 1998; Yao et al., 2014). *C. trachomatis* produces branched-chain fatty acids like *Staphylococcus aureus* (Parsons et al., 2011). Isoleucine is converted to 2-methylbutyryl-CoA by the branched-chain α-ketoacid dehydrogenase complex (Bkd). 3-Ketoacyl-ACP synthase III (FabH) is predicted to prime fatty acid synthesis using 2-methylbutyryl-CoA, as well as acetyl-CoA. Malonyl-CoA is generated from acetyl-CoA by the acetyl-CoA carboxylase complex (AccABCD), and converted into malonyl-ACP by malonyl-CoA:ACP transacylase (FabD). Malonyl-ACP is used for the two-carbon elongation of the growing fatty acid by FabH to initiate FASII, and by 3-ketoacyl-ACP synthase II (FabF) in subsequent elongation steps to make 3-ketoacyl-ACP. The 3-ketoacyl-ACP is reduced by 3-ketoacyl-ACP reductase (FabG) to 3-hydroxyacyl-ACP, which is then dehydrated by 3-hydroxyacyl-ACP dehydratase (FabZ) into *trans-2*-enoyl-ACP. *C. trachomatis* is a Gram-negative bacterium, and long-chain 3-hydroxyacyl-ACP is extracted from the FASII pathway for lipo-oligosaccharide synthesis (Rund et al., 1999; Heine et al., 2003, 2007). The *trans-2*-enoyl-ACP is reduced by enoyl-ACP reductase (FabI) in the last step of each elongation cycle to make acyl-ACP. All the enzymes in *C. trachomatis* FASII are highly similar (*e*-value < 1*e*^−40^) to their orthologs found in other free-living bacteria such as *E. coli* and *S. aureus*. The one exception is the predicted enoyl-ACP reductase FabI (*Ct*FabI), which is more closely related to the FabI found in the plastids of plants and Apicomplexan parasites like malaria (Yao et al., 2014).

### Initiation of Phospholipid Assembly by *C. trachomatis*

Bacterial phospholipid synthesis is initiated by the extraction of acyl-ACP of the appropriate length from the elongation cycle by two acyltransferases to synthesize phosphatidic acid, the key precursor to all phospholipids (Stephens et al., 1998; Yao et al., 2015a). The genome of *C. trachomatis* encodes two acyltransferases responsible for the successive acylation of *sn*-glycerol-3-phosphate (G3P) (**Figure 2**, Acyltransfer). The G3P acyltransferase in *C. trachomatis* (PlsE) is more related to the soluble G3P acyltranferase found in plant plastids (Turnbull et al., 2001) than the characterized bacterial membrane-bound G3P acyltransferases, PlsY and PlsB (Lightner et al., 1983; Lu et al., 2006; Yao and Rock, 2013). PlsE is established as a *bone fide* G3P acyltransferase by the biochemical characterization of purified PlsE. These experiments show that PlsE utilizes acyl-ACP as the acyl donor (Yao et al., 2015a).

The second step in the pathway is catalyzed by PlsC, 1-acyl-G3P acyltransferase (**Figure 2**). *Ct*PlsC is homologous to the well-characterized bacterial PlsCs, and complements *E. coli plsC*(Ts) mutants (Yao et al., 2015a). These enzymes are known to function as molecular rulers that select a specific acyl-ACP chain length for incorporation into the 2-position (Robertson et al., 2017). *Ct*PlsC exhibits this same property, and has a substrate selectivity like *Sa*PlsC in preferring 15:0-ACP. In complemented *plsC*(Ts) *E. coli* strains, *Ct*PlsC inserts 14:0 into the 2-position (like *Sa*PlsC), consistent with *C. trachomatis* phospholipids possessing 15:0 almost exclusively in the 2-position (Yao et al., 2015a). The presence of 15:0 provides a distinct molecular signature for all phospholipid molecular species produced by the *de novo C. trachomatis* biosynthetic pathway.

### Diversification of Phospholipid Headgroups

*Chlamydia trachomatis* is predicted to encode for the pathways necessary to synthesize phosphatidylglycerol (PG), cardiolipin (CL), and phosphatidylethanolamine (PE) from phosphatidic acid (**Figure 2**, Phospholipid) (Stephens et al., 1998; Yao et al., 2015a). These genes and phospholipid species are widely-distributed in free-living Gram-negative bacteria such as *E. coli* (Parsons and Rock, 2013). Phosphatidic acid is converted by CDP-diacylglyerol synthase (CdsA) into CDP-diacylglycerol. CDP-diacylglycerol is converted into phosphatidylserine by phosphatidylserine synthase (PssA), and then decarboxylated by phosphatidylserine decarboxylase (Psd) to make PE, the major phospholipid species in *C. trachomatis*. CDP-diacylglycerol is also converted into phosphatidylglycerol phosphate by phosphatidylglycerophosphate synthase (PgsA). The terminal phosphate of phosphatidylglycerolphosphate is removed by phosphatidylglycerophosphatase (Pgp) to make PG. Two PGs are ligated together by CL synthase to make CL.

Phospholipid compositional analysis of *C. trachomatis* infected HeLa cells showed significant increases in PE, PG, and CL composition compared to uninfected cells (Yao et al., 2015a). All the phospholipid molecular species of the new PE, PG, and CL produced in *Chlamydia*-infected cells contain 15:0, meaning that they were derived from phosphatidic acid produced by PlsE/PlsC (Yao et al., 2015a). *C. trachomatis* extracted and purified with the nonionic, non-denaturing detergent Nonidet P-40 was composed of ca. 85% PE/PG/cardiolipin containing branched-chain fatty acids and 15% host PC/sphingomyelin (Yao et al., 2015a). The PE molecular species contain 15:0, while the host PC molecular species did not (Yao et al., 2015a). The results of these experiments show that the bulk of *C. trachomatis* membrane is composed of chlamydial synthesized phospholipids containing chlamydial synthesized branched-chain fatty acids. The potential importance/function of the associated host lipids is discussed later in this review.

### *C. trachomatis* Does Not Modify Host Phospholipids

Several labs have concluded that host phospholipids such as PE and PC are deacylated and then reacylated with *C. trachomatis* derived branched-chain fatty acids (Wylie et al., 1997; Su et al., 2004; Soupene et al., 2015; Recuero-Checa et al., 2016; Soupene and Kuypers, 2017). However, there is no evidence that this inferred lipid modification pathway exists *in vivo* because the phospholipid molecular species analysis of *C. trachomatis* infected HeLa cells do not detect any of these putative products (Yao et al., 2015a). Branched-chain fatty acids are detected only in the phospholipid species predicted to be synthesized by *C. trachomatis*, and are not found in phospholipid species synthesized exclusively by the host, such as PC (Yao et al., 2015a). The only potential host phospholipid that may be modified by 15:0 reacylation is PE. Isotopically labeled ethanolamine is incorporated only into host PE species in *C. trachomatis*-infected HeLa cells because the *C. trachomatis* biosynthetic pathway produces PE from serine (via PS), not ethanolamine (Dowhan, 1997). The pool of PE labeled by ethanolamine in the *C. trachomatis*-infected HeLa cells did not contain 15:0, and had the same acyl-chain composition as the PE in uninfected host cells (Yao et al., 2015a). The idea that the host PS is used for PE synthesis (Soupene and Kuypers, 2017) is ruled out because host-derived PE molecular species are not found in purified *C. trachomatis* (Yao et al., 2015a). Phospholipid molecular species analysis clearly demonstrates that host PE is not deacylated/reacylated with branched-chain fatty acids.

### *C. trachomatis* 2-Acyl-GPE Acyltransferase/Acyl-ACP Synthetase System

Fatty acid synthesis is an energy and material intensive process, and bacteria encode the ability to incorporate exogenous fatty acids into their phospholipids (Yao and Rock, 2015, 2017b). Two mechanisms for incorporating exogenous fatty acids have been characterized in Gram-negative bacteria (**Figure 1**). First, exogenous fatty acids are converted into acyl-ACP by an acyl-ACP synthetase. The resulting acyl-ACP can be used by the acyltransferases for phosphatidic acid synthesis or further elongated by FASII (Jiang et al., 2006, 2010; Yao et al., 2016a). Second, exogenous fatty acids are converted into acyl-CoA by an acyl-CoA synthetase (Black et al., 1992; Yao et al., 2016a). The resulting acyl-CoA cannot be elongated by FASII, but can be used for phosphatidic acid synthesis (if the bacteria encode an acyltransferase capable of using acyl-CoA) or broken down via β-oxidation (if the bacteria encode for a β-oxidation pathway) (Yao and Rock, 2013). *C. trachomatis* lacks a β-oxidation pathway and uses acyl-ACP as the acyl donor for its acyltransferases. A third mechanism is fatty acid kinase that exists in Gram-positive bacteria that have a PlsY acyltransferase (Lu et al., 2006; Parsons et al., 2014), and is not present in *C. trachomatis*.

**FIGURE 3:**
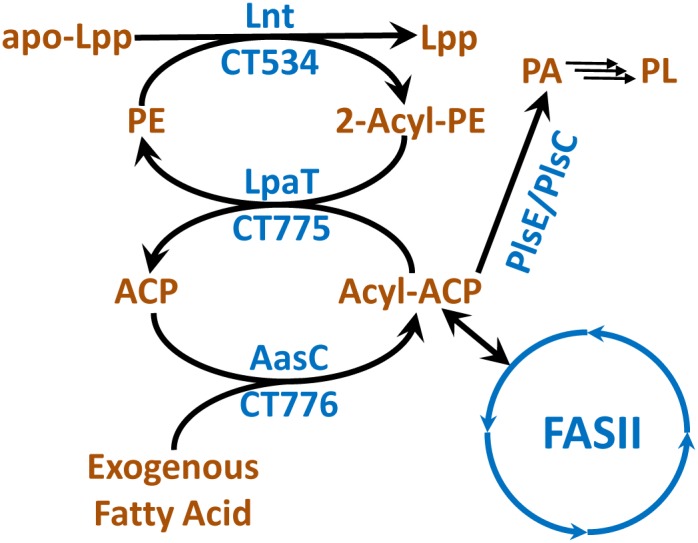
Fatty acid activation and lysophospholipid recycling in *C. trachomatis*. The amino-terminus of bacterial lipoproteins (apo-Lpp) are acylated by the transfer of fatty acids from the 1-position of PE to generate 2-acyl-glycerophosphoethanolamine (2-acyl-GPE) and acylated lipoproteins (Lpp). Other phospholipid species can also be used in the reaction. The 2-acyl-GPE is reacylated by LpaT using acyl-ACP. Acyl-ACP is derived from FASII or by the activation of exogenous fatty acids by AasC.

*Chlamydia trachomatis* is predicted to encode for the two domains of the *E. coli* acyl-ACP synthetase/2-acylglycerolphosphoethanolamine (GPE) acyltransferase bifunctional protein as two consecutive, but separate genes (**Figure 3**). In *E. coli*, this bifunctional gene is designated *aas*, and functions to reacylate 2-acyl-GPE formed by Lnt (apo-lipoprotein:phospholipid transacylase) during lipoprotein biogenesis with an acyl-ACP (Jackowski and Rock, 1986). The lipoprotein synthesis pathway is widely distributed in Gram-negative bacteria, and *C. trachomatis* lipoproteins have the same lipid modifications as observed in *E. coli* (Everett et al., 1994; Neff et al., 2007). The genes encoding the lipid modification pathway are present (*lgt*, CT252; *lsp*, CT408; *lnt*, CT534). Thus, it is reasonable to conclude that the split acyl-ACP synthetase (AasC) and 2-acyl-GPE acyltransferase (LpaT) system of *C. trachomatis* also supports phospholipid re-cycling during lipoprotein maturation.

There are differences between the *E*. *coli* and *C. trachomatis* systems that expand the function of this system in *Chlamydia*. In *E. coli*, the carboxy-terminal acyl-ACP synthetase activity is physically linked to the amino-terminal 2-acyl-GPE acyltransferase activity (**Figure 1**). Acyl-ACP generated by the *E. coli* acyl-ACP synthetase domain can only be used by the amino-terminal domain 2-acyl-GPE acyltransferase, and cannot enter FASII or be used by the other cellular acyltransferases (Rock and Cronan, 1979; Cooper et al., 1989; Hsu et al., 1991). In *C. trachomatis*, the two domains are present as two separate genes (**Figure 3**). This alternate genetic structure separates *C. trachomatis* acyl-ACP synthetase (AasC) from the acyltransferase (LpaT) suggesting that AasC may release acyl-ACP that could be used by FASII and all cellular acyltransferases, instead of being metabolically channeled exclusively to LpaT. Biochemical characterization of purified AasC shows that it converts fatty acid only to acyl-ACP (not acyl-CoA) with the same preference for 1-position fatty acids as the *E. coli* system (Yao et al., 2015b). The heterologous expression of AasC enables the incorporation of exogenous fatty acids into the phospholipids in an *E. coli* strain deficient in exogenous fatty acid activation showing that AasC generates acyl-ACPs from exogenous fatty acids that is available to cellular acyltransferases (Yao et al., 2015b). LpaT is biochemically characterized as a 2-acyl-lysophosholipid acyltransferase, and *Ct*LpaT also complements *E. coli* mutants lacking the 2-acyl-GPE acyltransferase (Yao et al., 2015b). Lysophospholipids rapidly undergo acyl chain migration, and these substrates are always present as a mixture of 1-acyl and 2-acyl lysophospholipids (Pluckthun and Dennis, 1982). Thus, one must analyze the positional distribution of acyl chains in the phospholipid product to be certain about the structure of the lysophospholipid substrate used by the enzyme.

The analysis of *C. trachomatis*-infected HeLa cells showed that about 8% of the chlamydial PE correspond to a PE molecular species containing one 15:0 and one 18:1 unsaturated fatty acid (Yao et al., 2015a). Polyunsaturated fatty acids that are abundant in mammals are not detected in *C. trachomatis* phospholipids. Because *C. trachomatis* does not encode for the ability to synthesize unsaturated fatty acid (Stephens et al., 1998), this result suggests that AasC activates host fatty acids in the cell infection model. This hypothesis is tested by labeling *C. trachomatis* infected HeLa cells with isotopically labeled, medium chain fatty acids (12:0 and 14:0), and observing whether these fatty acids are elongated and incorporated into the *C. trachomatis* phospholipids (Yao et al., 2015b). *C. trachomatis* could elongate and incorporate these exogenous, isotopically labeled fatty acids into its phospholipid species, showing that the *C. trachomatis* AasC is activating exogenous fatty acids during the infection cycle that were utilized by FASII or acyltransferases. Polyunsaturated fatty acids are abundant in the host cell, but are not found in *C. trachomatis* phospholipids consistent with the observed substrate selectivity for saturated fatty acids, and some reactivity with oleate by the AasC.

## Host Phospholipids in the Chlamydial Life Cycle

### Host Lipid Trafficking Is Essential for the Growth and Maintenance of the Chlamydial Inclusion

The chlamydial inclusion grows significantly in size during the course of the infection, from holding the single internalized EB at the beginning of the infection cycle to taking up the majority of the internal volume of the host cell at the end of the infection cycle (Elwell and Engel, 2012). The significant increase in the surface area of this chlamydial inclusion membrane during the chlamydial cell cycle necessitates some way to grow this membrane. It has not been possible to define the membrane composition of the inclusion directly because it is challenging to isolate this compartment and to separate the associated *C. trachomatis* from the membrane. Immunofluorescence studies demonstrated the presence of host lipid species in inclusion membrane (Hackstadt et al., 1995; Carabeo et al., 2003; Beatty, 2008; Elwell et al., 2011), and there is emerging evidence that host and chlamydial proteins are found in the inclusion membrane (Beatty, 2008; Mital et al., 2013; Weber et al., 2017). The landmark paper by Hackstadt et al. (1995) demonstrates that trafficking of the host lipids is essential for the growth of the chlamydial inclusion. Fluorescently labeled sphingolipids synthesized by the host cell from C_6_-NBD-ceramide co-localized with the inclusion membrane and *C. trachomatis*, showing that sphingolipids and potentially other host lipids are trafficked into the inclusion. Adding Brefeldin A, an inhibitor of vesicular trafficking, stops this co-localization. The disruption of this trafficking by Brefeldin A led to smaller chlamydial inclusions and premature lysis of chlamydial inclusion (Elwell et al., 2011), demonstrating that the trafficking of host lipids is essential for the growth and maintenance of the chlamydial inclusion. Further research shows that several chlamydial inclusion membrane proteins are essential for the trafficking of host lipids, and the depletion of these proteins also leads to the premature lysis of the chlamydial inclusion (Weber et al., 2017). These experiments show that the chlamydial inclusion membrane proteins recruit host phospholipids via vesicular trafficking pathways to the chlamydial inclusion.

There are extensive investigations in literature demonstrating the transport of other host phospholipids, cholesterol, lipid droplets, high-density lipoproteins, and even host organelles into the chlamydial inclusion as well as the role these transport plays in the infection cycle (Cocchiaro et al., 2008; Elwell et al., 2011; Cox et al., 2012, 2016; Bastidas et al., 2013; Agaisse and Derre, 2014; Al-Zeer et al., 2014; Boncompain et al., 2014; Saka et al., 2015). Inhibition of these pathways usually lead to decreased *C. trachomatis* infectious titers. These trafficked lipids might be other components of the chlamydial inclusion membrane. *C. trachomatis* also actively incorporates host fatty acids for *de novo* phospholipid synthesis, and some of these pathways, such as the lipid droplet trafficking pathway, might deliver the host fatty acids through the chlamydial inclusion membrane. Given that *C. trachomatis* is auxotrophic for a variety of metabolic precursors, it is very clear that there must be many mechanisms to traffic these metabolic precursors into the chlamydial inclusion. Detailed discussion of host and pathogen interactions can be found in literature (Elwell and Engel, 2012; Bastidas et al., 2013) and is beyond the scope of this review.

### Are Host Lipids an Essential Component of the *C. trachomatis* Membrane?

The fluorescently labeled lipid analogs co-localized with the replicating reticulate bodies (Hackstadt et al., 1995), suggesting that trafficked host lipids are potentially essential constituents of the *C. trachomatis* membrane. One challenge in interpreting experiments using fluorescently labeled lipids is recognizing that fluorescent groups such as NBD replacing the fatty acid in the fluorescently labeled lipid analogs are significantly bulkier and more hydrophilic. Therefore, these probes have increased solubility compared to the actual lipid, making fluorescently labeled lipid analogs capable of diffusing to places where the actual lipids would not. A variety of different analysis confirms that host phosphatidylcholine and sphingolipids are indeed co-purified at low abundance with *C. trachomatis* even under stringent purification (Yao et al., 2015a; Cox et al., 2016). Some authors have concluded that this proves that these lipids are essential structural components of the *C. trachomatis* membrane (Cox et al., 2016). However, these same host lipids are observed using the same purification procedures in the absence of *C. trachomatis* infection, raising genuine concern about whether the low amounts of host lipids in the purified *C. trachomatis* preparation are actually components of the bacterial membrane (Yao et al., 2015a).

Whether host phospholipids are essential constituents of the *C. trachomatis* membrane or just associated with *C. trachomatis* could be definitely settled by determining if *C. trachomatis* can replicate in absence of these host lipids. This experiment is impossible to execute in practice because *C. trachomatis* grows intracellularly with direct contact to the host derived chlamydial inclusion (Mirrashidi et al., 2015; Weber et al., 2017). The low titers observed through inhibiting the various host lipid trafficking pathways do not address whether host lipids are an essential component of the *C. trachomatis* membrane because the growth of the chlamydial inclusion is also required for the continued replication of the reticulate body (Elwell et al., 2011; Cox et al., 2016). Regardless, inhibition of host lipid tracking pathways is an effective method of disrupting the *C. trachomatis* life cycle and reducing the generation of infectious elementary bodies.

## Inhibition of Fatty Acid Synthesis in *C. trachomatis*

Fatty acid and phospholipid synthesis is one of the four major conserved biosynthetic pathways in living organisms, and enzymes in the pathway have a long history of being investigated as novel antibiotic targets (Yao and Rock, 2016, 2017a). FabI and FabH were identified and validated as essential gene targets in bacteria by target-based discovery campaigns conducted by GlaxoSmithKline in the 1990s (Payne et al., 2007). FabI have received extensive research as an antibiotic target, with the FabI inhibitor AFN-1252 having recently passed phase 2 clinical trials as a narrow spectrum, *S. aureus* specific antibiotic (Kaplan et al., 2012, 2013; Flamm et al., 2015; Hunt et al., 2015; Hafkin et al., 2016).

### FASII Is Essential in *C. trachomatis*

Because FabI is the rating-controlling step in fatty acid elongation (Heath and Rock, 1995), *Ct*FabI was biochemically characterized and structurally elucidated. *Ct*FabI catalyzed the reduction of *trans-2*-enoyl-ACP and complements an *E. coli fabI*(Ts) strain, validating its function as an enoyl-ACP reductase (Yao et al., 2014). The *Ct*FabI was inhibited by triclosan and AFN-1252, two well-described FabI inhibitors, at low micromolar affinity (Yao et al., 2014). The *Ct*FabI•NAD(H)•AFN-1252 ternary complex was elucidated using X-ray crystallography, and shows that the *Ct*FabI forms a similar ternary complex with AFN-1252 as the previously characterized ternary complex structure with *S. aureus* FabI (Yao et al., 2014). AFN-1252 has cleared two Phase II clinical efficacy trials, and lacks off-target effects against human cells (Hunt et al., 2015; Hafkin et al., 2016). Therefore, the *C. trachomatis*-infected HeLa cell model was treated with AFN-1252 to understand how the inhibition of *Ct*FabI and FASII effects the intracellular growth of *C. trachomatis*. AFN-1252 caused a dose dependent inhibition of the replication of *C. trachomatis* correlating with selective inhibition of *C. trachomatis* fatty acid synthesis, demonstrating that the inhibition of chlamydial FASII inhibits the growth and replication of *C. trachomatis* (Yao et al., 2014). AFN-1252 blocked the transition of *C. trachomatis* at a reticulate body like state when AFN-1252 was added at infection and prevented the generation of any infectious units (Yao et al., 2014). AFN-1252 also caused decreased infectious titers when added post infection over the entire replication cycle (Yao et al., 2014). These experiments show that FASII is required for the transition of elementary bodies into the metabolically active reticulate bodies as well as the replication of the reticulate bodies. These data also directly validate *Ct*FabI as an anti-chlamydial drug discovery target, and suggest that targeting other enzymes in FASII would be effective as well.

FabI inhibition by AFN-1252 blocked the replication of *C. trachomatis* despite the abundance of exogenous host fatty acids in the *C. trachomatis* infected HeLa cell model and the ability to activate exogenous fatty acids with AasC (Yao et al., 2015b). This result appears counterintuitive at first glance, but it is common for FASII inhibitors to block cell growth in the presence of ample extracellular fatty acids (Parsons et al., 2011). The explanation for this finding is that blockage of the elongation cycle leads to the accumulation of the acyl-ACP intermediate used by the inhibited FASII step. This results in free ACP being sequestered as the short-chain thioester biosynthetic intermediate, making it unavailable for exogenous fatty acid activation. The depletion of free ACP prevents the incorporation of exogenous fatty acids by acyl-ACP synthetase, making it impossible to bypass FASII inhibition. This result is observed with many other bacterial species that also encode for exogenous fatty acid incorporation pathways, but cannot bypass FASII inhibition (Yao and Rock, 2015; Yao et al., 2016a).

There has been debate in literature about whether environmental fatty acids can enable the emergence of fatty acid auxotrophic versions of common pathogens like *S. aureus* (Morvan et al., 2016), how fit these auxotrophic bacteria are (Morvan et al., 2016), whether these auxotrophic bacteria are present in nature (Gloux et al., 2017), and what all this means for FASII targeting antibiotics (Morvan et al., 2017; Yao and Rock, 2017b). Loss of function in the fatty acid initiation genes leads to fatty acid auxotrophic *S. aureus* (Parsons et al., 2011, 2013). These mutants bypass FASII inhibition by utilizing exogenous fatty acids for phospholipid synthesis instead. These mutants occur at high frequency (1 × 10^−6^) from any mutation that causes loss of function in the initiation module (Parsons et al., 2011, 2013). High levels of exogenous fatty acids are required for the growth of these mutants, and their growth rate in liquid culture significantly lags their wild type counterpart (Parsons et al., 2011, 2013). The fatty acid auxotrophic mutants appear to have a significant growth defect *in vivo* as well. The Δ*accD* mutant does not proliferate at all in the bacteremia model (Parsons et al., 2013). The *fabD* gene truncation mutant has decreased growth in the tail vein infection model compared to wild type (Morvan et al., 2016). Certain point mutations to FASII genes can enable *S. aureus* to become more resistant to FASII therapeutics, but without becoming fatty acid auxotrophic and not able to bypass FASII inhibition. These mutants have minimal growth defects (Morvan et al., 2016) as they are able to synthesize fatty acids endogenously. The issue of resistance in antibiotic discovery will be discussed in the next section. The environmental prevalence of these auxotrophic *S. aureus* mutants was estimated by screening *S. aureus* on media containing the FASII inhibitor triclosan and fatty acid supplementation (Gloux et al., 2017). Screening found that 7% of clinical and veterinary samples contained colonies that grew on the selective media and requires fatty acid supplementation. This result was interpreted as a wide prevalence of fatty acid auxotrophic *S. aureus* in the environment (Gloux et al., 2017). Whether this “prevalence” represents a persistent population of fatty acid auxotrophic *S. aureus* in the environment, or transient mutants that arise from the high frequency loss of function mutations is unclear. Regardless, the evidence is clear that fatty acid auxotrophic versions of *S. aureus* can arise frequently under selection pressure even if they are not in the environment already. Environment prevalence or fast emergence of *S. aureus* capable of bypassing FASII inhibition is predicted to cause significant failures to FASII therapeutics if these mutants don’t have significant fitness defects (Morvan et al., 2016). However, AFN-1252 was highly successful in clinical trials even in patients with significant comorbidities (Hafkin et al., 2016), consistent with the observations that fatty acid auxotrophic *S. aureus* has significant growth defects (Parsons et al., 2011, 2013). We have noted that the endogenous synthesis of branched-chain fatty acids appear essential for the proper growth and proliferation of bacterial species containing *anteiso*15:0 (Yao and Rock, 2015, 2017a,b).

Bypassing FASII inhibition through fatty acid auxotrophy is not predicted to be a viable mechanism in *C. trachomatis*, although more experiments are needed. First, *C. trachomatis* could not bypass FASII inhibition even when AFN-1252 is added 8 h post infection when there is a large population of growing *C. trachomatis* and host cell protein synthesis is inhibited (Yao et al., 2014). This result suggests that fatty acid auxotrophic mutants must have fitness defects given the high frequency that mutations causing fatty acid auxotrophy is predicted to occur. Second, *C. trachomatis* encodes for a fully functional fatty acid synthesis system (Stephens et al., 1998; Yao et al., 2014) despite being in an exogenous fatty acid rich environment and actively trafficking/incorporating host fatty acids over the growth phase (Yao et al., 2015b). The conservation of FASII suggests an essential role given that *C. trachomatis* has a reduced genome that has replaced biosynthetic capabilities with host trafficking in other essential pathways (Stephens et al., 1998). The essentiality of FASII may be related to branched-chains fatty acids or lipooligosaccharides (Yao and Rock, 2015).

### FabI as a Pathogen Selective Antibacterial Target

The well-reasoned “multi-target” hypothesis proposes that broad-spectrum, monotherapeutic antibiotics in clinical use today target multiple gene targets (Silver, 2007, 2011; Yao and Rock, 2017a) because point mutations in the gene target allows the fast development of resistance against single-target antibiotics in bacteria. Because broad-spectrum antibiotics usually only have micromolar affinity as a compromise to achieve the broad-spectrum, a small increase in resistance is sufficient to render the antibiotic ineffective in the clinic. Multi-targeting overcomes this hurdle because the probability of developing resistance against several gene targets is multiplicative (i.e., 1 × 10^−9^ vs. one target, 1 × 10^−18^ vs. two targets, 1 × 10^−27^ for three targets, etc) (Yao and Rock, 2017a). Unfortunately, only three known sets of gene targets are structurally close enough to allow for efficient multi-targeting: penicillin binding proteins, ribosomes, and DNA gyrases (Silver, 2007).

The FabI inhibitor, AFN-1252, is currently under clinical trials as an example of a pathogen selectively antibiotic. Rather than compromising affinity to achieve a broad spectrum of activity, AFN-1252 is designed to have low nanomolar affinity against *S. aureus* FabI (Yao et al., 2013). Missense mutations to the *S. aureus* FabI can significantly increase resistance to AFN-1252, from 4 ng/ml against FabI(WT) to 250 ng/ml against FabI(M99T) and 500 ng/ml against FabI(Y147H) (Yao et al., 2013). However, additional rounds of selection did not produce mutants with higher levels of resistance. The FabI(M99T, Y147H) double mutant was nonviable. These results show that even the most resistant mutant found via the selection experiment is still susceptible to therapeutically achievable doses of AFN-1252 (Kaplan et al., 2013), suggesting that high affinity, pathogen selective inhibitors might prove to be therapeutically effective antibiotics. AFN-1252 is the only antibiotic following this paradigm in clinical trials, having recently passed phase II trials (Hafkin et al., 2016). Its development will validate or disprove the pathogen selective inhibitor idea.

A narrow spectrum is a feature of FabI inhibitors because many bacteria, such as *Streptococcus pneumonia*, encode for a different structural class of enoyl-ACP reductases called FabK that is resistant to FabI inhibitors (Heath and Rock, 2000; Marrakchi et al., 2003). Although pharmaceutical companies traditionally strived for broad spectrum antibiotics due to economic incentives, narrow spectrum antibiotics have gained increasing interest (Maxson and Mitchell, 2016; Spaulding et al., 2017). Recent advances in microbiome research demonstrates that broad spectrum antibiotic treatment decimates the commensal microbiota, and is linked to adverse effects such as increased secondary infections in the short term (Bohnhoff and Miller, 1962; Buffie et al., 2012) and increased chances of metabolic diseases in the long term (Blaser and Falkow, 2009; Cho et al., 2012; Modi et al., 2014; Boursi et al., 2015). The normal treatment for *C. trachomatis* infection is high-dosage Azithromycin, which is known to significantly alter the microbiome (Million et al., 2013; Parker et al., 2017). The narrow spectrum FabI inhibitor, AFN-1252, has been shown to minimize collateral damage to the microbiome, and would decrease these potential negative side effects (Yao et al., 2016b). A chlamydial selective FabI inhibitor is particularly alluring for novel anti-chlamydial therapeutics, because FabI is a validated target and minimizing collateral damage to the microbiome would be an improvement over existing therapeutics. Furthermore, AFN-1252 had reasonable affinity against the *Ct*FabI in the cellular model, suggesting that AFN-1252 is a valid starting structure for additional rounds of structural optimization to generate a high affinity *C. trachomatis* selective inhibitor and potentially speeding up the discovery process (Yao et al., 2014).

## Author Contributions

JY and CR conceived, drafted, and revised the manuscript.

## Conflict of Interest Statement

The authors declare that the research was conducted in the absence of any commercial or financial relationships that could be construed as a potential conflict of interest.
